# From sinusitis to blindness: an unusual case of Pott’s puffy tumor with bilateral optic atrophy

**DOI:** 10.1093/omcr/omag050

**Published:** 2026-05-10

**Authors:** Marouane Jidal, Soufiane Ibat, Youssra Kramchi, Achraf Jeddab, Mohammed Rabi Andaloussi, Khalil Mounir, Hicham Balkhi

**Affiliations:** Department of Surgical Intensive Care Unit, Mohammed V Military Teaching Hospital, FAR Avenue, Hay Riad, Rabat, Morocco; Department of Surgical Intensive Care Unit, Mohammed V Military Teaching Hospital, FAR Avenue, Hay Riad, Rabat, Morocco; Department of Surgical Intensive Care Unit, Mohammed V Military Teaching Hospital, FAR Avenue, Hay Riad, Rabat, Morocco; Department of Surgical Intensive Care Unit, Mohammed V Military Teaching Hospital, FAR Avenue, Hay Riad, Rabat, Morocco; Department of Surgical Intensive Care Unit, Mohammed V Military Teaching Hospital, FAR Avenue, Hay Riad, Rabat, Morocco; Department of Surgical Intensive Care Unit, Mohammed V Military Teaching Hospital, FAR Avenue, Hay Riad, Rabat, Morocco; Department of Surgical Intensive Care Unit, Mohammed V Military Teaching Hospital, FAR Avenue, Hay Riad, Rabat, Morocco

**Keywords:** Pott’s puffy tumor, sinusitis, cerebral empyema, cavernous sinus thrombosis, optic atrophy, case report

## Abstract

**Introduction:**

Pott’s puffy tumor (PPT) is a rare complication of sinusitis and is defined as a subperiosteal abscess with frontal bone osteomyelitis. Intracranial and orbital complications occur frequently.

**Case Presentation:**

A 17-year-old Senegalese male presented with febrile headache and right frontal swelling. Computed tomography (CT) revealed frontal empyema, orbital cellulitis, pansinusitis, venous sinus thrombosis, and septic pulmonary emboli. Despite antibiotic treatment, he required sinus exclusion, ethmoidectomy, and neurosurgical drainage. Cultures grew *Haemophilus influenzae*, Pseudomonas spp., and Neisseria spp.. Postoperatively, the bilateral optic atrophy led to irreversible blindness. Follow-up MR angiography revealed regression of the infection and sinus recanalization. Antibiotics and anticoagulants were discontinued after three months; the patient remained blind and awaited cranioplasty.

**Discussion:**

PPT often causes epidural or subdural abscesses; however, blindness is exceedingly rare.

**Conclusion:**

PPT should be suspected in adolescents with persistent frontal swelling. Early multidisciplinary management is vital for preventing severe morbidities.

## Introduction

Pott’s puffy tumor (PPT) is an uncommon yet serious complication of sinusitis, characterized by the development of a subperiosteal abscess in association with frontal bone osteomyelitis. First described by Sir Percivall Pott in the 18th century, the condition predominantly affects adolescents, a vulnerability attributed to incomplete pneumatization of the frontal sinus and the presence of a highly developed diploic venous network [1, 2].

Despite significant advances in diagnostic imaging, recognition of PPT is frequently delayed, thereby increasing the risk of severe, potentially life-threatening complications [3].

Herein, we report an exceptional case of PPT presenting with a rare outcome of bilateral blindness.

## Case report

A 17-year-old Senegalese male was admitted with facial cellulitis following a 10-day history of febrile headaches and progressive right frontal–periorbital swelling. Prior empirical therapy initiated at another institution with ceftriaxone, gentamicin, and metronidazole was ineffective, allowing the infection to progress and necessitating referral for further evaluation and management.

**Figure 1:**
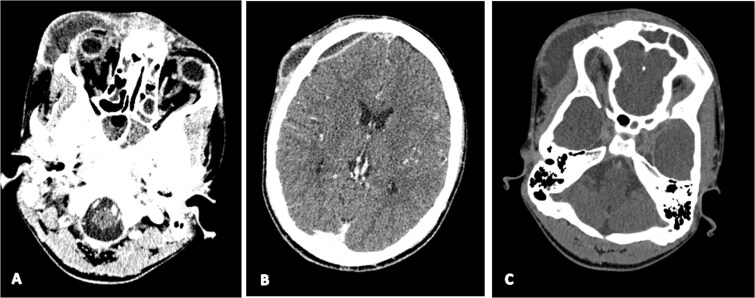
Axial contrast-enhanced brain CT showing right orbital cellulitis (A), right frontal empyema (B), and bilateral cavernous sinus thrombosis (C).

**Figure 2:**
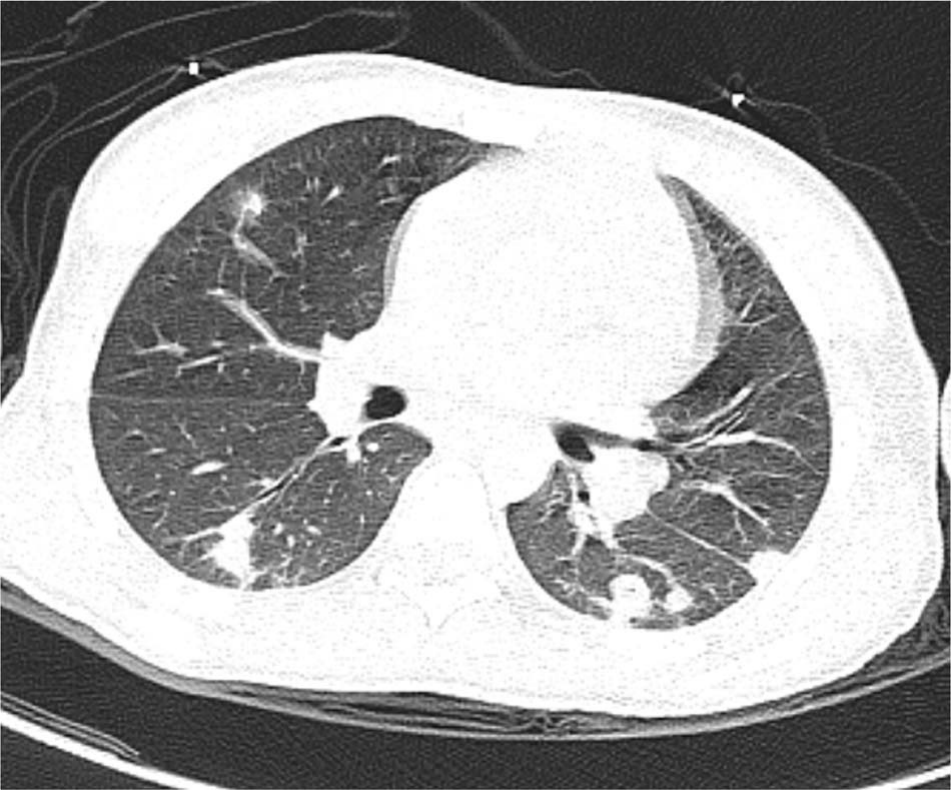
Axial chest CT (parenchymal window) demonstrating septic emboli.

**Figure 3:**
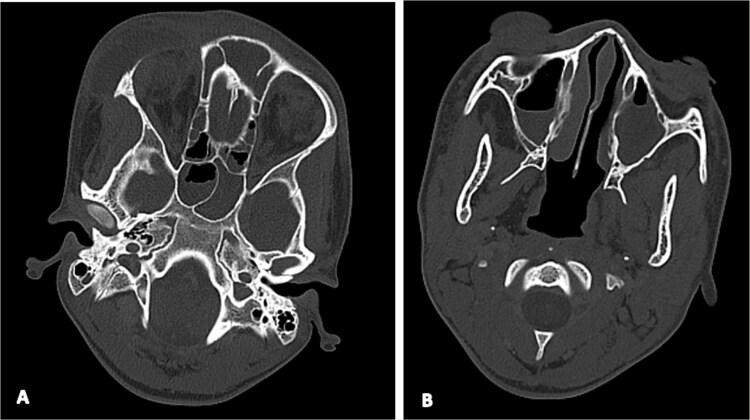
Axial brain CT (bone window) showing ethmoido-frontal sinusitis (A) and bilateral maxillary sinusitis (B).

On admission, the patient exhibited marked right frontal swelling, periorbital cellulitis, meningeal irritation signs, and an altered level of consciousness with a Glasgow Coma Scale score of 11/15 (due to inability to open eyes from periorbital edema, not altered consciousness). Contrast-enhanced computed tomography revealed extensive disease, including frontal cerebral empyema, orbital cellulitis, pansinusitis, and lateral sinus thrombosis extending into the right internal jugular vein, along with multiple septic pulmonary emboli and pericardial effusions (Figs 1, 2 and 3). Laboratory investigations demonstrated leukocytosis (16 400/μl) and; elevated CRP (107 mg/l), and PCT (5.1 μg/l) levels.

Cerebrospinal fluid analysis via lumbar puncture revealed neutrophilic pleocytosis with Gram-positive cocci. Frontal drainage yielded pus containing gram-positive cocci and bacilli, and multiplex PCR subsequently identified *Haemophilus influenzae*. Empirical broad-spectrum antimicrobial therapy, including meropenem, vancomycin, metronidazole, and fluconazole, was initiated along with therapeutic anticoagulation.

**Figure 4:**
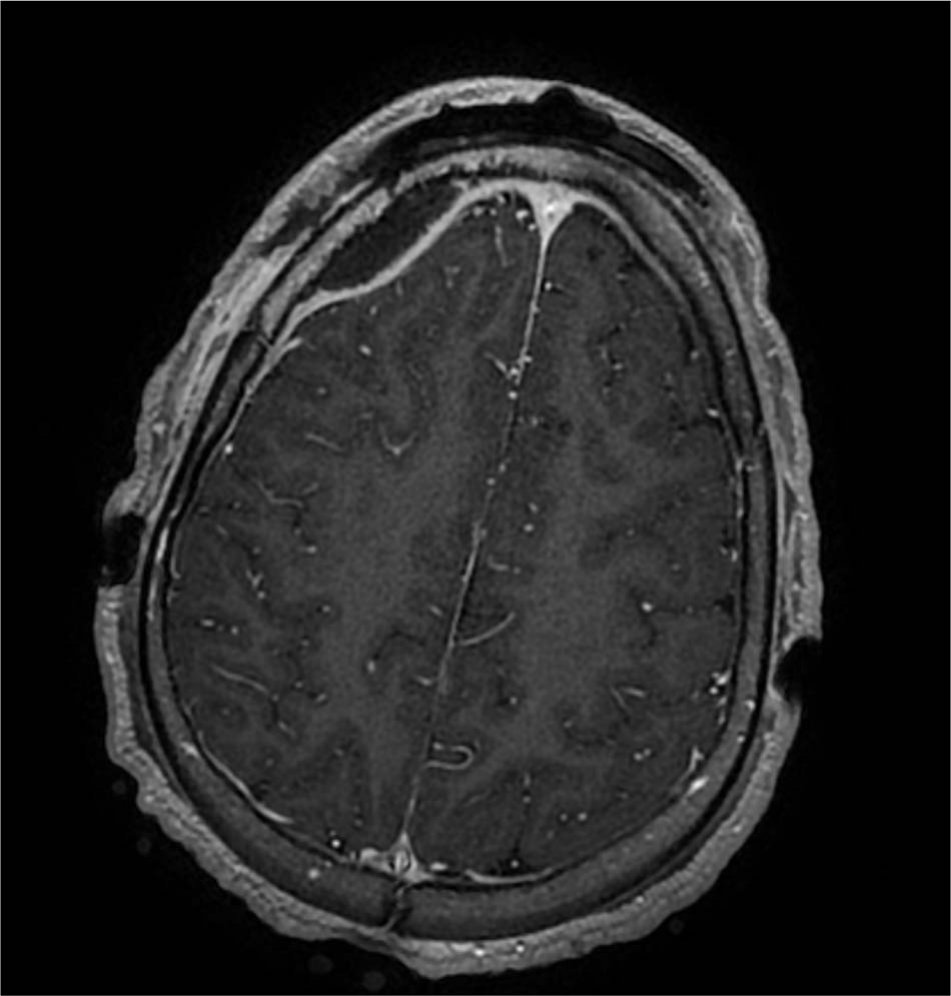
Axial contrast-enhanced T1-weighted MRI at postoperative day 10 showing persistent cerebral empyema.

Owing to persistent fever and ongoing infection, the patient underwent endoscopic frontal sinus exclusion and bilateral ethmoidectomy. After reduction of the palpebral edema under anti-edematous therapy, purulent chemosis and complete loss of light perception were documented; these findings were considered to reflect pre-existing optic nerve involvement that could not be assessed earlier because the eyelids could not be opened. On day 10, he experienced seizures associated with a marked increase in inflammatory markers levels (CRP, 160 mg/l). Magnetic resonance imaging revealed persistent cerebral empyema and ongoing venous sinus thrombosis (Fig. 4), prompting neurosurgical drainage and debridement of frontal osteitis. Intraoperative cultures subsequently showed *Pseudomonas* spp. and *Neisseria*. After three weeks of intravenous antimicrobial therapy, the patient remained afebrile and clinically stable, allowing de-escalation to an oral regimen consisting of a fluoroquinolone and amoxicillin–clavulanate, with close monitoring of body temperature, C-reactive protein, and procalcitonin levels.

**Figure 5:**
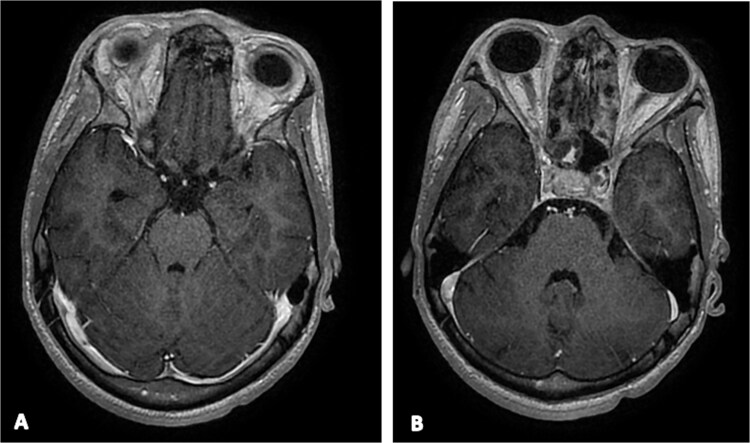
MR venography at 1 month demonstrating patency of the right lateral sinus (A) and recanalization of the cavernous sinuses (B).

**Figure 6:**
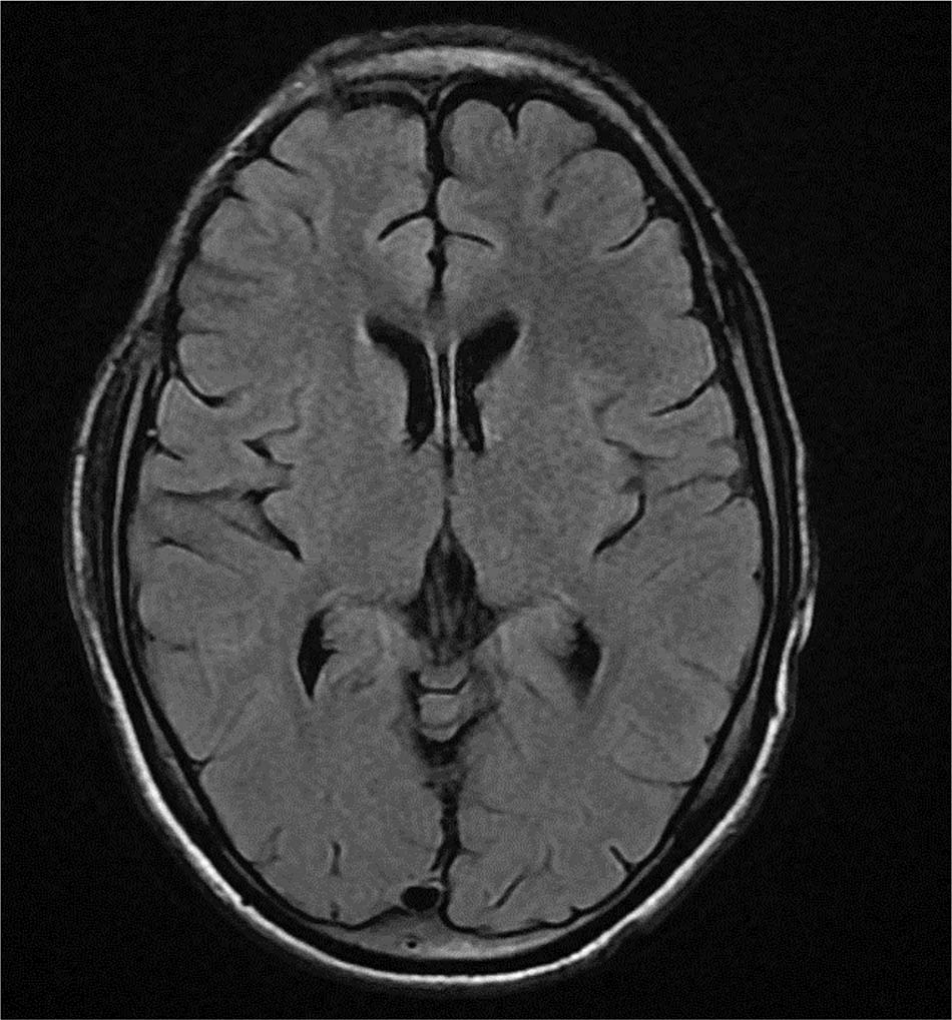
Axial FLAIR MRI at 1 month postoperatively showing the right frontal craniectomy flap with complete resolution of the cerebral empyema.

Follow-up MR venography at one month demonstrated regression of the empyema and recanalization of the affected venous sinuses (Figs 5, 6). Ophthalmological evaluation confirmed bilateral optic atrophy. After three months, antimicrobial and anticoagulation therapies were discontinued, and antiepileptic treatment was maintained. The patient remains blind and scheduled for delayed cranioplasty. The overall clinical course is summarized in the timeline (Table 1).

**Table 1:** Timeline of the patient’s clinical course, including symptom onset, diagnostic workup, surgical procedures, antimicrobial therapy, and follow-up.

Time point (relative to admission)	Clinical events and key findings
Pre-admission period (≈ Day −10 to Day 0)	Onset of febrile headache with progressive right frontal and periorbital swelling, followed by empirical treatment at an outside facility with intravenous ceftriaxone, gentamicin, and metronidazole, without clinical improvement.
Day 0—Admission	Admission for facial cellulitis and suspected complicated sinusitis. Contrast-enhanced CT: frontal cerebral empyema, orbital cellulitis, pansinusitis, lateral sinus thrombosis extending to the right internal jugular vein, septic pulmonary emboli, and pericardial effusion. Broad-spectrum IV meropenem, vancomycin, metronidazole, and fluconazole started with therapeutic anticoagulation.
Day 2	Persistent fever and inflammatory response despite antibiotic therapy. Endoscopic frontal sinus exclusion and bilateral ethmoidectomy performed.
Day 2–3	Reduction of palpebral edema allows ophthalmologic assessment: purulent chemosis and complete bilateral loss of light perception documented, consistent with severe optic nerve involvement.
Day 10	Onset of seizures with a marked increase in inflammatory markers. MRI: persistent frontal empyema.
Day 11	Neurosurgical drainage of the frontal empyema and debridement of frontal osteitis. Intraoperative cultures growing *Pseudomonas* spp. and *Neisseria* spp.
Day 21	After 3 weeks of intravenous antibiotic therapy, treatment was switched to oral fluoroquinolone and amoxicillin–clavulanate.
1-month follow-up	MR venography: regression of intracranial infection and recanalization of the affected venous sinuses. Ophthalmologic evaluation confirming bilateral optic atrophy and irreversible blindness.
3-month follow-up	Discontinuation of antimicrobial and anticoagulation therapies. Patient remains blind and is scheduled for delayed cranioplasty; antiepileptic treatment continued.

## Discussion

Pott’s puffy tumors exhibit a marked male predominance (male-to-female ratio approximately 3:1) and primarily affect adolescents and young adults, with a mean age of 17.4 years [4]. The pathophysiology involves the spread of infection either through septic thrombophlebitis of the diploic veins or via direct extension after trauma [2, 3].

The most commonly implicated pathogens include *Staphylococcus aureus*, *Staphylococcus epidermidis*, various streptococcal species (approximately 44%), and anaerobic bacteria, often of dental origin [3, 5]. Notably, cultures may yield no growth if antimicrobial therapy is initiated before sampling [3].

Clinically, patients typically present with frontal swelling, headaches, and fever [4]. The presence of neurological deficits may indicate intracranial extension, which occurs in up to 85% of cases [1, 5]. The most frequent intracranial complications are epidural abscesses (47%), subdural empyema (25%), and intracerebral abscesses (12%) [3]. Ophthalmologic involvement is common but generally reversible, with only 5% of cases requiring surgical intervention [4]. Bilateral blindness, as observed in our patient, remains extraordinarily rare, with one single previous report documented in a systematic review [4].

The cavernous sinus is a key venous plexus of the middle cranial fossa that drains the orbit, face, and anterior skull base, and lies in close relation to cranial nerves III, IV, V1, V2, and VI, as well as the internal carotid artery [6]. Because the cerebral venous system is essentially valveless, infections arising from the paranasal sinuses or orbit can rapidly extend to the cavernous and lateral sinuses, as illustrated in our patient with extensive sinusitis, orbital cellulitis, and venous sinus thrombosis. Vision loss in cavernous sinus thrombosis may result from central retinal or ophthalmic vein occlusion, carotid artery involvement, or toxic and ischemic optic neuropathy [7]. In our case, the sudden bilateral loss of light perception followed by optic atrophy is most consistent with a predominantly ischemic optic neuropathy on a background of severe orbital cellulitis and intracranial venous congestion. The combination of raised intraorbital pressure due to purulent chemosis, thrombosis of the cavernous and lateral sinuses, and possible intracranial hypertension related to the frontal empyema likely led to an irreversible mixed compressive–ischemic injury of both optic nerves.

Accurate diagnosis relies primarily on contrast-enhanced CT of the brain and paranasal sinuses, with MRI serving as a complementary modality when intracranial complications are suspected [3].

Management necessitates prolonged administration of broad-spectrum antibiotics with adequate bone penetration, typically over 6–12 weeks, combined with surgical drainage of abscesses and debridement of the necrotic bone. Effective treatment mandates a multidisciplinary approach, involving otolaryngology, neurosurgery, ophthalmology, and intensive care specialists [3, 5].

In our patient, the mechanism of bilateral optic atrophy was probably multifactorial. The presence of severe orbital cellulitis with purulent chemosis suggests a component of compressive optic neuropathy due to raised intraorbital pressure. In addition, the documented thrombosis of the lateral and cavernous venous sinuses may have contributed to ischemic optic neuropathy through impaired venous outflow, reduced perfusion of the optic nerves, and intracranial venous congestion. Finally, intracranial hypertension related to the frontal empyema and venous sinus thrombosis may have exacerbated axonal injury at the level of the optic nerves and chiasm. The rapid and irreversible loss of light perception, followed by the appearance of optic atrophy, is consistent with a combination of compressive and ischemic mechanisms rather than an isolated, slowly progressive neuropathy.

## Conclusion

Pott’s puffy tumor remains an uncommon yet potentially life-threatening complication of sinusitis in adolescents. Timely recognition, comprehensive imaging, and prompt multidisciplinary intervention are essential to minimize morbidity and prevent serious sequelae. Bilateral blindness, as illustrated in this case, represents one of the rarest and most devastating outcomes, underscoring the critical importance of early diagnosis and aggressive management.
